# In the Brain, It Is Not All about Sugar

**DOI:** 10.3390/neurosci5020016

**Published:** 2024-06-19

**Authors:** Bernardo C. Antunes, Tomás Mateus, Vanessa A. Morais

**Affiliations:** Instituto de Medicina Molecular-João Lobo Antunes, Faculdade de Medicina, Universidade de Lisboa, 1649-028 Lisbon, Portugal; bernardo.antunes@medicina.ulisboa.pt (B.C.A.); tomasmateus@medicina.ulisboa.pt (T.M.)

**Keywords:** brain, mitochondria, fatty acid metabolism, synapse

## Abstract

The maintenance of energetic homeostasis relies on a tight balance between glycolysis and mitochondrial oxidative phosphorylation. The case of the brain is a peculiar one, as although entailing a constant demand for energy, it is believed to rely mostly on glucose, particularly at the level of neurons. Nonetheless, this has been challenged by studies that show that alternatives such as lactate, ketone bodies, and glutamate can be used as fuels to sustain neuronal activity. The importance of fatty acid (FA) metabolism to this extent is still unclear, albeit sustaining a significant energetic output when compared to glucose. While several authors postulate a possible role of FA for the energetic homeostasis of the brain, several others point out the intrinsic features of this pathway that make its contribution difficult to explain in the context of neuronal bioenergetics. Moreover, fueling preference at the synapse level is yet to be uncovered. In this review, we discuss in detail the arguments for and against the brain usage of FA. Furthermore, we postulate that the importance of this fuel may be greater at the synapse, where local mitochondria possess a set of features that enable a more effective usage of this fuel source.

## 1. Introduction

Cells maintain their energetic homeostasis by metabolizing sugars, amino acids, and fatty acids, shuttling them into the tricarboxylic acid (TCA) cycle. This will generate reducing agents NADH and FADH_2_, which will carry electrons to the electron transport chain (ETC), located in the inner mitochondrial membrane (IMM). This generates an electron flow through the ETC, which will prompt the pumping of proton into the intermembrane space (IMS). In the last step of the process, to produce energy, protons then flow down their electrochemical gradient through F1F0-ATP synthase, also known as mitochondrial complex V or ATP synthase, to generate ATP. Although oxidative phosphorylation is the largest source of cellular ATP, the potential energy generated by the ETC is also harnessed for biosynthetic purposes [1]. In addition to mitochondrial oxidative phosphorylation (OXPHOS), cells rely on glycolysis for energy production, especially in conditions where the oxygen supply is scarce [2]. This process occurs in the cytosol, where glucose is converted into pyruvate via a series of metabolic reactions, being nevertheless a relatively inefficient way of ATP generation compared with OXPHOS [2]. However, which fuel source a cell prefers to use at certain time point and which mechanism dictates the switch between preferential fuel sources remains to be clarified. The brain is a very peculiar case in this extent, as despite being a very energy-“hungry” organ, it prefers to metabolize mostly glucose to sustain its energetic needs, via both glycolysis and OXPHOS [3], while additional fuels such as fatty acids and ketone bodies are thought to be used in particular circumstances only [4]. However, evidence for the metabolism of lipids by the brain has started to increase [5,6,7,8]. Aligned with a high demand for energetic supply, the fact that the brain contains the second highest concentration of lipids in the human body [9] makes the importance of this fuel undeniable, with the increased peculiarity of its role in defining neuronal structures, which is vital for the compartmentalization of the many neuronal signaling processes [10]. Moreover, the increased body of evidence showing an association between dysfunction in lipid metabolism and brain pathologies [10,11] reinforces the importance of proper scrutiny of this fuel source in the context of the brain.

In this review, we discuss the relevance of fatty acid metabolism as a fuel for brain and neuronal bioenergetics, particularly at the level of neuronal periphery and synapse. We also highlight how neuronal compartmentalization may be key for understanding different fuel preferences within neurons.

## 2. The Brain and Its Fuels

The human brain has a high energy demand, consuming up to 74% of oxygen in newborns, and about 20% to 23% in adults, daily [12].

Herein, energy is used mostly for neurotransmission, axonal or dendritic transport, the synthesis of neurotransmitters, and, to a lower extent, protein synthesis. Mitochondria are the main contributors to ATP generation (more than 90%) via OXPHOS, representing the largest portion of the ATP turnover performed during presynaptic and postsynaptic signaling. Cell-wise, neurons are the most energy-demanding cells, whereas the energy consumption of astrocytes amounts to about 5–15% of the total energy requirement of the brain. The energy metabolism of neurons is mainly aerobic respiration, and that of astrocytes is mainly anaerobic glycolysis [12]; however, evidence of the metabolic flexibility of astrocytes was recently showed, as cells were able to metabolize extracellular pyruvate to lactate and alanine or via mitochondrial OXPHOS, the latter mediated by the action of mitochondrial pyruvate carrier [13]. Additionally, evidence of some astrocytic capacity for fatty acid oxidation (FAO) has started to arise [11].

In terms of fueling preference, the current dogma states that glucose is the main energy substrate for neurons and glia cells [12]. Generally, energy reserves are low in brain tissue and are limited to a small amount of glycogen, which is exclusively stored in astrocytes [12], as the glycolytic capacity of neurons is lower, due to the lack of activity of the glycolysis-promoting enzyme 6-phosphofructo-2-kinase/fructose 2,6-biphosphatase, isoform 3, PFKFB [14]. Astrocytic glycolysis then converts glucose to lactate, which will be supplied to neurons via monocarboxylate transporters MCT1, 2, and 4, fueling synaptic activity [15]. This will lead to the further stimulation of glycolysis in astrocytes, maintaining this fuel cycle that shows evidence of the importance of metabolic coupling between neurons and glial cells. Additionally, the use of lactate as a fuel enables neurons to allocate higher amounts of glucose for nicotinamide adenine dinucleotide phosphate (NAPDH) generation through the pentose phosphate pathway, crucial for the maintenance of neuronal anti-oxidative status [14]. Nevertheless, recent evidence shows that neurons also rely on aerobic glycolysis, particularly at the soma, with elevated expression levels of key glycolytic enzymes such as PKM2 in the cell body when compared with nerve terminals [16]. When PKM2 was inhibited, preventing the conversion of pyruvate onto lactate, this caused a switch from glycolysis to oxidative metabolism with increased oxidative damage levels at neuronal somata [16].

As more layers of complexity are found on the subject of brain metabolism, this dogma of strict glucose dependence by neurons is also being revisited. Additional fuels such as pyruvate were recently shown to be important regulators of synaptic vesicle cycle homeostasis [17]. Inhibition of the mitochondrial pyruvate carrier (MPC), responsible for transporting pyruvate into mitochondria, led to disruption of the spatial dynamics of synaptic vesicle endo-exocytosis [17]. The work of Divakaruni and colleagues added further evidence of neuronal fuel flexibility, showing a shift from glucose to glutamate metabolism upon MPC inhibition [18]. Glutamate is produced in mitochondria, from the catabolism of glutamine by the enzyme glutaminase (GLS). Similar to pyruvate, glutamate allows for both energy production and the refilling of mitochondrial metabolites (which can be converted into α-ketoglutarate by glutamate dehydrogenase, sustaining the maintenance of TCA cycle intermediates [1]). The authors also observed that upon MPC inhibition, neuronal stimulation led to lower cell death levels, possibly related to lower excitotoxic effects related with lower levels of glutamate released [18].

In addition to the scenarios described above, certain conditions compel the brain to also use ketone bodies (KBs) and fatty acids (FAs) as fuels, although the extent of the physiological relevance of the metabolism of fatty acids, in particular, is still a matter of debate [7].

## 3. Fatty Acid Fueling

Fatty acids (FAs), and dietary fats in particular, typically comprise 30–40% of energy intake and consist mostly of long-chain fatty acids esterified in triacylglycerols [19]. Importantly, the metabolic breakdown of FAs renders a profit that is able to surpass glucose by more than twofold (in the case of long-chain fatty acids such as palmitate, a 16-carbon saturated FA) [1].

Structural-wise, FAs are composed of hydrogenated carbons with a carboxyl moiety at the alpha carbon, and present fundamental constituents of the biology of the cell. Based on the number of double bonds found within the carbon chain, FAs are organized in three groups: saturated fatty acids (SAFAs), monounsaturated fatty acids (MUFAs), and polyunsaturated fatty acids (PUFAs) [20]. SAFAs, characterized by the absence of double bonds, are fully hydrogenated, and essential participants in maintaining the integrity of cellular membrane structures (primary element of phospholipids and sphingolipids). Additionally, SAFA can act as post-translational modifiers [20]. Palmitic acid, or palmitate, for instance, can modify cysteine (or less frequently serine and threonine) residues in a process termed palmitoylation. The palmitoylation of proteins often facilitates docking to membranes; many transmembrane proteins also undergo palmitoylation. This process has been shown to trigger important pathways involved in the activation of innate and adaptive immune systems [21]. In the brain, protein palmitoylation was shown to facilitate axonal and dendritic growth during neuronal development, as well as regulating the traffic and docking of proteins between the plasma membrane and intracellular compartments (Golgi apparatus, endoplasmic reticulum (ER), and recycling endosomes) during plastic changes that occur in the synapses of neuronal networks [22]. The role of palmitoylation in a pathological context has been shown in Parkinson’s disease, with the accumulation of α-synuclein as a downstream effect of the palmitoylation of vesicle-trafficking protein Synaptotagmin-11 (Syt11) [23]. Failure to maintain a balance of palmitoylation/depalmitoylation has also been shown to contribute to the onset of other neurodegenerative pathologies such as Huntington’s disease (HD) [22].

MUFAs, presenting a single double bond, are also primary constituents of cellular membrane structures and glycerolipids.

PUFAs, which can have up to six double bonds in their carbon chain, are usually found in the phospholipids of membrane structures, and may also act as precursors to a variety of lipid signaling molecules. Interestingly, mammals are unable to synthesize de novo PUFAs, since they lack the desaturase enzymes required for producing the limiting substrate for PUFA synthesis. Therefore, PUFAs are considered essential fatty acids that must be acquired from the diet [20]. Docosahexaenoic acid (DHA), an essential omega-3 PUFA, is mostly localized in neuronal membranes, and is involved in processes such as neurogenesis, synaptogenesis, neuronal differentiation, and neurite outgrowth. DHA can also attenuate the inflammatory response of microglia by decreasing the production of proinflammatory cytokines, such as tumor necrosis factor-α (TNF-α) and interleukin 6 (IL-6), among others [4]. Arachidonic acid (AA), an omega-3 PUFA, is actively involved in neuronal development, and is also important in the context of cell division, myelin enrichment, and the preservation of hippocampal cell membranes, which confers a protective role in neuronal aging [24]. Carbon chain length itself can also differentiate FAs and impact on their function, as the chain length of fatty acids can modify cellular membrane properties, such as the fluidity and formation of microdomains and signaling platforms, and alter their susceptibility to cell death or survival [20].

## 4. Fatty Acid Synthesis and Oxidation

### 4.1. Fatty Acid Synthesis

The balance of FA synthesis and degradation is vital for the maintenance of homeostasis in the organism. A full description of fatty acid synthesis may be found in [20]. This process occurs mainly in the cytosol of cells as a way of storing energy from carbohydrate-derived carbon precursors. Briefly, acetyl-CoA (produced by ATP citrate lyase or ACLY), is metabolized by acetyl-CoA carboxylase 1 (ACACA), a rate-limiting enzyme of this pathway, culminating with the production of malonyl-CoA, limiting reagents of this process. From this point onwards, fatty acid synthetase (FASN) will add malonyl-CoA to growing acyl-chains, producing different saturated FAs depending on chain length (palmitate comprises about 80–90% of FASN’s total product). Additionally, these synthesized fatty acids can be converted into triglyceride molecules for storage. The expression of FASN is mostly observed in the liver, brain, and abdominal adipose tissue, where energy storage is important for cell survival during periods of physiological or pathological stress [20].

Aside from the cytoplasmatic process characterized by the well-known and well-studied enzyme FASN, described above, mitochondria also harbor a spatially and genetically distinct fatty acid synthesis pathway (mtFAS) [25]. In contrast to FASN, which is a very large protein that contains several domains and encompasses all the enzymatic activities necessary for FAS condensed in a single polypeptide chain, the mtFAS pathway comprises at least six enzymes all encoded by separate genes. This process sequentially adds two carbons to a growing acyl chain per cycle. Among the several differences between mtFAS and cytoplasmic FAS, FASN produces mostly palmitate, whereas mtFAS appears to have at least two major products. One known product of mtFAS is an eight-carbon saturated fatty acid, octanoate, that can be further converted to lipoic acid. This important cofactor is required for the catalytic activity of a number of mitochondrial enzymes, such as pyruvate dehydrogenase and α-ketoglutarate dehydrogenase, branched chain amino acid dehydrogenase, the H protein of the glycine cleavage system, and 2-oxoadipate dehydrogenase. Notably, the loss of lipoic acid synthesis and/or failure to efficiently transfer lipoic acid to its target proteins was shown to be lethal in mice [25]. In addition to octanoate, mtFAS can produce longer acyl chains of at least 14 carbons, whose functions are yet to be better understood. Importantly, Nowinski and colleagues found that loss of the mtFAS pathway leads to a profound impairment in the assembly of the mitochondrial electron transport chain machinery, with the attendant consequences on metabolism and cell behaviors [25].

The importance of FA synthesis has already been shown in the context of brain homeostasis. Astrocytic FA synthesis, for instance, is essential for neuronal differentiation during development [26]. This is achieved through the accumulation of oleic acid, a MUFA, promoting differentiation in neurons. Oleic acid can also be used by oligodendrocytes to synthesize sphingomyelin, stimulating myelination [26]. The role of FA synthesis on microglia is twofold: SAFA synthesis apparently favors activation of a proinflammatory phenotype, while PUFA biosynthesis promotes an anti-inflammatory profile. MUFA synthesis influence, on the other hand, seems to depend on the disease context [26]. mtFAS may also be of therapeutic value, as lipoic acid efficiently downregulates proinflammatory processes such NF-kB translocation, controlling the release of cytotoxic cytokines and free radicals. Lipoic acid was also reported to reduce demyelization and axonal loss [26].

### 4.2. Fatty Acid Oxidation

Although it was long assumed that the cellular uptake of fatty acids was mostly the result of simple diffusion, it is now known that other molecular mechanisms can facilitate this process. While membrane-associated proteins, such as FABPpm and CD36, can serve as acceptors for fatty acids to increase their concentration at the cell surface, promoting and enhancing fatty acid diffusion events, and facilitate their transport across the phospholipid bilayer (uptake by facilitated diffusion), other transporters such as FATP1 can also mediate the membrane crossing of a minority of fatty acids [19].

Once in the cytosol, acyl-CoA synthetases activate fatty acids to form acyl-coenzyme A (CoA) esters, which then can be degraded in a process generally termed fatty acid oxidation (FAO). FAO can be carried out by mitochondria (dietary long-chain FAs), as well as peroxisomes (branched- and very-long-chain FAs) [4], although the latter can only shorten, but not fully degrade, FAs into acetyl-CoA. In the extent of peroxisomal FAO, carnitine does not participate in the uptake of FA, but rather in the export of the chain-shortened product to mitochondria [4].

#### 4.2.1. Carnitine Shuttle

Mitochondrial FAO requires the import of acyl-CoA. Nevertheless, as the mitochondrial membrane is impermeable to certain acyl-CoAs, a mechanism known as the carnitine shuttle is needed to mediate this transport, and this is particularly important in the case of long-chain fatty acids [27]. Although some tissues can synthesize carnitine, it is mostly obtained through diet, being transported across the plasma membrane by the organic cation transporter OCTN2 (SLC22A5) [28].

The first mediator of this transport is the rate-limiting carnitine-palmitoyl transferase I (CPT1), which conjugates acyl-CoA molecules to carnitine. Acylcarnitines are then transported across the highly impermeable inner mitochondrial membrane by the carnitine-acylcarnitine translocase (CACT), being re-processed to free acyl-CoAs, which are then released into the mitochondrial matrix via the action of carnitine-palmitoyl transferase 2 (CPT2). Moreover, free carnitine is transported back to the cytoplasm [27]. Medium- and short-chain acyl-CoAs enter mitochondria directly.

#### 4.2.2. Fatty Acid β-Oxidation

It is inside the mitochondrial matrix that the degradation of acyl-CoAs occurs, a process also known as fatty acid β-oxidation. Briefly, this is characterized by the sequential metabolism of acyl-CoAs by four enzymatic steps catalyzed by enzymes varying in chain length specificity: acylCoA dehydrogenases (ACADs), enoyl-CoA hydratases, L-3-hydroxyacyl-CoA dehydrogenases, and 3-ketoacyl-CoA thiolases [27]. The products of each four-step cycle constitute an acyl-CoA shortened by two carbon atoms, an acetyl-CoA molecule, and one nicotinamide adenine dinucleotide (NADH) and one flavin adenine dinucleotide (FADH_2_) as electron carriers (or reducing equivalents). The resulting acyl-CoA is further metabolized in another cycle of FAO; the acetyl-CoA can enter the citric acid cycle, and electron carriers deliver the electrons to the electron transport chain [29].

As it happens, fatty acid synthesis and oxidation are tightly correlated mechanisms, one serving as a key regulator of the other, orchestrated by the action of the 5′-adenosine monophosphate (AMP)-activated protein kinase (AMPK). The key players in this cross-regulation are the FAO rate-limiting enzyme CTP1 and its allosteric inhibitor Malonyl-CoA (synthesized by acetyl-CoA carboxylase—ACC), involved in the process of FAS, as described above. In the context of energetic shortage or exercise, AMPK phosphorylates and inactivates ACC to reduce malonyl-CoA concentration, promoting the entry of long-chain acyl-CoA into the mitochondria (via CPT1 activity) for β-oxidation to restore energy balance [30].

## 5. Ketone Body Metabolism

In certain conditions, such as periods of low glucose availability in the blood or intense physical activity, FAs can also be mobilized from fat reserves into the liver to produce ketone bodies (KBs). This metabolic pathway, termed ketogenesis, produces three types of molecules: β-hydroxybutyrate (β-HB), acetoacetate (AcAc), and acetone. β-HB and AcAc are important energy sources for hardworking organs such as the brain, heart, and skeletal muscle [31]. During this metabolic state, KBs are therefore seen as an alternative and effective energy source to glucose.

To generate ATP, KBs need to metabolize inside mitochondria (matrix), through the ketolysis pathway [31]. β-Hydroxybutyrate can be oxidated to acetoacetate, which undergoes two enzymatic reactions to be converted into acetyl-CoA, the final product of the ketolysis pathway. The acetyl-CoA molecules can then be incorporated into the TCA cycle, enabling the generation of ATP and NADH. Subsequently, these NADH molecules can supply electrons to the ETC, enabling the production of additional ATP through OXPHOS.

Ketone bodies are also accounted as important fuels during brain development when circulating glucose levels are unable to match the demand for energy and the high rate of macromolecular biosynthesis [3]. The fact that newborns are in permanent ketosis, regardless of feeding status and the fact that the brain uptake of KBs is up to five times faster than in adults, highlights the importance of this fuel for the developing brain. KBs can serve as both energetic substrates via mitochondrial OXPHOS, as well as substrates for the anabolism of structural metabolites such as cholesterol, which represents 20% of total brain lipids [3].

## 6. Fatty Acid Metabolism in the Brain

**Figure 1:**
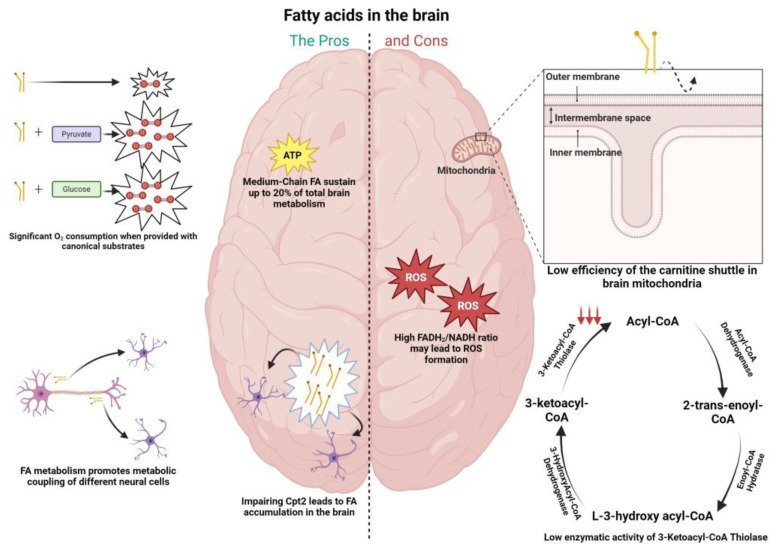
Pros (**left column**) and cons (**right column**) of fatty acid metabolism in the brain. (**Left**) Fatty acid fueling of neuronal mitochondria leads to significant levels of oxygen consumption when provided in conjunction with substrates as pyruvate and glucose; medium-chain fatty acids (octanoate) were shown via ^13^C nuclear magnetic resonance spectroscopy to contribute up to 20% of total rat brain oxidative energy production; fatty acid accumulation was shown in the brains of Drosophila when inducing loss of function in the carnitine shuttle enzyme (CPT2), with the effect more pronounced at the level of astrocytes; fatty acid metabolism was shown to promote metabolic coupling between neurons and astrocytes. Specifically, peroxidized lipids were shuttled from neurons to astrocytes, promoting fatty acid oxidation in the latter. (**Right**) The efficiency of mitochondrial fatty acid import (CPT1) is lower in the brain; activity of the β-oxidation enzyme 3-ketoacyl-CoA thiolase present in brain mitochondria was shown to be low when compared with the same enzymes in other tissues known to deal with fatty acids (0.7% of that in heart mitochondria); the FADH_2_/NADH ratio is elevated in the context of palmitic acid (0.48), when compared to glucose (0.22) and ketone bodies (0.33), facilitating the leakage of electrons to molecular oxygen and generating greater oxidative damage, which can be harmful to neurons.

Fatty acid metabolism sustains an extensive energetic yield, making it of vital importance for several tissues in the organism such as the heart, skeletal muscle, and kidneys. Nevertheless, its importance has been questioned in the case of the brain, where it is still unclear the extent to which fatty acids can satisfy this organ’s energetic demands and homeostasis [4,7,12,14]. Visual examples of this dichotomic role can be found in Figure 1.

### 6.1. Cons to FA Metabolism in the Brain

Arguments that have been belittling the importance of fatty acid metabolism in the brain are based on an apparently limited capacity from brain mitochondria to import fatty acids through the carnitine shuttle (Figure 1), as well as on a restricted enzymatic capacity of brain mitochondria to oxidize this fuel, when compared to mitochondria from other tissues [12].

Tackling the first argument, some reports have localized CPT1c (a brain isoform of CPT1) in the endoplasmic reticulum, implying a role that focuses predominantly on a biosynthetic pathway rather than in the degradation of LCFAs [32]. Additionally, the supply of brain mitochondria with the non-esterified or carnitine derivatives of octanoic or palmitic acid did not translate in a different mitochondrial ROS generation, excluding a proclivity towards FAO, where usually a higher ROS generation is correlated with the degradation of carnitine derivatives. In contrast, liver mitochondria exhibit much higher ROS generation with the carnitine derivatives [12]. As for the second argument, it was shown that the rate of mitochondrial fatty acid oxidation by rat brain mitochondria is lower than in the heart or liver mitochondria, with l-palmitoylcarnitine as a substrate, ascribed to low specific activities of the β-oxidation enzymes present in brain mitochondria 3-ketoacyl-CoA thiolase (0.7% of that in heart mitochondria). Therefore, the low activity of 3-ketoacyl-CoA thiolase, has been identified as rate limiting for mitochondrial fatty acid degradation in the brain (Figure 1) [12,33]. Interestingly, the degradation of ketone bodies in the brain is more fine-tuned, correlated with the high activity of enzymes such as succinct thiolase, involved in the processing of acetoacetyl-CoA, formed from the ketone body acetoacetate [12]. Another argument showing the downside of the usage of fatty acids in the brain is related to the oxidative damage that this fuel may cause, particularly in neurons. The FADH_2_/NADH ratio, a measure of the energetic yield of a specific fuel, is elevated in the context of palmitic acid (0.48), when compared to glucose (0.22) and ketone bodies (0.33). This implies a higher production of NADH and FADH_2_, leading to greater competition between the two factors to donate electrons to Complex I of mitochondria, facilitating its leakage of electrons to molecular oxygen, and therefore the greater oxidative damage (Figure 1) [12]. The notion that the brain does not utilize FAs to sustain energy needs can also be attributed to the fact that the body, in circumstances of glucose shortage, relies on the production of KBs from fat reserves to sustain the brain’s energetic needs [34].

### 6.2. The Pros of FA Metabolism in the Brain

As opposed to the “cons” mentioned above in relation to FA metabolism, there is already evidence showing that this fuel may be more important for brain homeostasis than initially thought. A study from Ebert and colleagues showed via ^13^C nuclear magnetic resonance spectroscopy that octanoate (a medium-chain fatty acid) contributes up to 20% of total brain oxidative energy production (Figure 1) [5]. Schulz et al. [6] also showed that the adult brain in Drosophila is able to metabolize FAs (Figure 1). As mentioned above in relation to glucose metabolism, evidence has also been shown for the metabolic coupling between neurons and astrocytes in the context of fatty acid metabolism. Ioannou et al. showed that upon neuronal hyperactivity, peroxidized lipids are transferred from neurons to astrocytes, where they are further degraded through mitochondrial oxidation (Figure 1) [35]. To this extent, the medium-chain fatty acids decanoic acid and octanoic acid were able to potentiate lactate and ketone body formation in astrocytes, respectively, which can activate shuttle systems to provide nutrients for neighboring neurons [36]. Additionally, challenging the view that neuronal mitochondria in adult brain do not oxidize FAs, Panov and colleagues showed that isolated rat brain mitochondria utilize FAs as an energy source in astrocytes and neurons when furnished in association with other respiratory substrates, a situation that resembles the in vivo conditions (Figure 1) [7].

### 6.3. The Role of Fatty Acids in Brain Pathophysiology

The beneficial role of lipid metabolism in the brain has been questioned mainly due to the possible implication of the dysfunction in the metabolism of this fuel leading to several pathologies at the level of the brain. In the context of amyotrophic lateral sclerosis (ALS), the deficient capacity to handle glucose has led to speculation that alternate substrates such as lipids may be metabolized to maintain energy balance [10]. Studies in mouse models and ALS patients showed increased lipid catabolism and clearance to peripheral tissues, with concomitant increases in the markers of oxidative stress and lipid peroxidation in brain and spinal cord tissue [10]. Moreover, lipid peroxidation associated with the risk of oxidative damage has also been linked to neurodegenerative conditions such as Alzheimer’s disease (AD) and Parkinson’s disease (PD) [37].

Nevertheless, evidence for the protective role of lipids in the context of brain pathology can also be found. Observations of upregulated lipid metabolism upon the inhibition of glycolytic enzyme HK2 in the microglia of an AD mouse model revealed an attenuation of cognitive impairment linked to increased ATP production and a decrease in amyloid plaque burden [38]. Additionally, and opposed to what is observed under chronic high-fat-diet regimens (HFD), mice exposed to an acute HFD increased cerebrospinal fluid palmitate levels, triggering a metabolic switch in microglia characterized by elevated fatty acid β-oxidation, increased aerobic glycolysis, mitochondrial membrane activation and fission, and improved learning and memory [39]. Additional evidence for the importance of balanced lipid metabolism was shown by Liu and colleagues, where lipid droplet accumulation led to peroxidation and oxidative damage [40]. In this scenario, rescuing lipase function effectively delayed neurodegeneration [40]. Moreover, diets rich in monounsaturated FAs (e.g., the Mediterranean diet) have demonstrated protective effects at the level of neurodegeneration, slowing a risk of AD, and decreasing chronic inflammation levels in human glioblastoma [4].

## 7. Ketone Bodies in the Brain

In comparison with FAs, the role of KBs in the brain is less a subject of discussion. Indeed KBs are known to be metabolized by neurons to produce ATP [41]. Furthermore, their presence seems to be essential to assure the homeostasis of the brain’s energy levels [42]. However, their role in the brain is extremely dependent on the body’s metabolic state. When physiological glucose levels are present, their concentration is nearly negligible (ketogenesis is not stimulated in the liver) [42].

**Figure 2:**
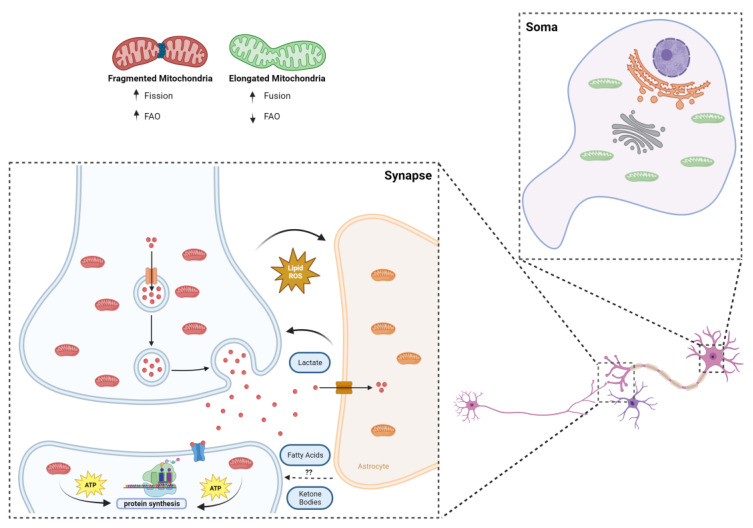
Synaptic mitochondria and the challenging environment of the synapse. The smaller size of synaptic mitochondria is a sine qua non condition for their inhabitance of synaptic terminals, but it can also mean greater propensity to deal with fatty acids: it was recently shown that more fragmented mitochondria are less sensitive to CPT1 inhibition; mitochondria located post synapse were shown to be the main source of local energy production for protein translation and synaptic plasticity, taking advantage of their “strategic” position within dendrites to supply the local neighborhood of synapses; metabolic coupling between neurons and astrocytes is vital in maintaining a healthy environment, whereby toxic lipids are transferred from neurons to astrocytes, and metabolites of importance for synaptic plasticity, such as lactate (and possibly ketone bodies and fatty acids), follow the opposite route.

The current state of the art recognizes the liver as a unique organ capable of producing KBs from FAs [43]. However, emerging evidence has pointed out that astrocytes might also be important KB producers. The work of Bryon Silva and colleagues revealed, using the Drosophila animal model, that astrocyte-like glial cells use their own lipid droplets to synthesize KBs [44], which are further oxidized by neurons and are crucial to sustain memory formation during periods of starvation. Another study showed that, in mice, white mater degeneration, a common hallmark of Alzheimer’s disease, is accompanied with increased levels of fatty acids, mitochondrial fatty acid enzymes, and the rise of ketone body levels in the brain [45]. They hypothesize that myelin lipids are catabolized into FAs which, in turn, are converted into KBs by astrocytes, therefore sustaining neuronal fueling as a compensatory mechanism for neuronal energy deficit [45]. This evidence suggests that KBs may play an important role in supporting neuronal energy requirements, particularly in states of disease or nutrient shortage. Astrocytes, by producing and delivering KBs to neurons, appear to be key contributors in meeting their energy demands (Figure 2).

## 8. Fatty Acids and Synaptic Mitochondria

Dwelling further into the fueling preference of the brain, synapses represent the most interesting objects of study in the discipline of metabolism, as it is here that the most energy-demanding processes in the brain take place [46]. Synapses are terminal compartments, isolated from neuronal soma in distances that can sometimes be measured in meters. Synaptic compartments are characterized by possessing mitochondria with a quite unique fingerprint, with the majority of presynaptic terminals occupied by mitochondria, while only 10–25% of postsynaptic compartments show the presence of these organelles [46]. Indeed, mitochondria are transported to pre-synaptic terminals in kinesin-mediated trafficking [47], occupying close to one-third of the volume of the presynaptic terminal, which implies an important role for these organelles in synaptic transmission [46]. In cortical neurons, for instance, evidence shows that mitochondrial capture occurs at presynaptic boutons, and organelle disruption leads to a significant decrease in branch growth, and ultimately, branch retraction, while boosting mitochondria function rescues this phenotype [48]. This role in axonal branching, as well as evidence that shows that mitochondrial impairment leads to deficits at the level of synaptic vesicle cycling and implicated in pathologies such as Leigh’s disease, imply that the presence of mitochondria in pre-synaptic terminals is key for the regulation of neuronal homeostasis [49].

Since the first studies took place in the 1970s [50,51], trying to separate synaptic from non-synaptic mitochondria, an increasing volume of information about this specific population has been unveiled through the years. In summary, synaptic mitochondria show distinct enzymatic expression and activity [52,53,54], as well as a different lipidome and proteome [55,56,57], also differing from their non-synaptic counterparts in shape and size [46]. The latter aspect is crucial for the “colonization” of synaptic terminals, since the average neuronal mitochondrion would be too large to fit in a presynaptic ending and to pass through axons [46]. Indeed, Li et al. showed that a dominant negative form of Dynamin-related protein 1 (Drp1), a key orchestrator of mitochondrial fission, reduced the mitochondrial localization in dendrites after depolarization [58]. Functionally, one can argue that the unique properties of synaptic mitochondria may be directly linked to the homeostasis of the whole synaptic compartment. For instance, synaptic mitochondria were shown to be more sensitive to the inhibition of electron transport chain protein complexes and to the damaging influence of calcium [46]. Synaptic degeneration is an early hallmark of neurodegeneration; therefore, the vulnerability of synaptic mitochondria to physiological stresses may suggest its direct implication in the pathogenesis of various brain diseases. Fueling-wise, a recent study by Rangaraju and colleagues showed, via high-resolution imaging, that mitochondria are the main source of local energy production for protein translation and synaptic plasticity (Figure 2), taking advantage of its “strategic” position within dendrites to supply the local neighborhood of synapses, with direct causes in the process of memory formation [59]. As for the fueling preference per se, the case of neurons and synapses is still unclear.

## 9. Conclusions and Future Perspectives

Hypothetically, although the importance of glucose in neuronal and synaptic function goes without saying, the energetic demand and pressures inherent to the environment that surrounds synaptic terminal raises the question of whether additional fuel sources may or not be of use to keep synaptic circuitry functioning properly. Astrocytes, for instance, have been shown to be quite flexible in the substrates used, with competences at the level of glycolysis and FAO [35,60]. If, on the one hand, they can take away peroxidized lipids that can potentially damage neurons, on the other hand they may provide lactate to fuel synaptic plasticity [15,61], and who knows if beneficial fatty acids and ketone bodies may not travel this road as well (Figure 2). Additionally, the unique fingerprint of synaptic mitochondria may enable this population to properly deal with high-content energy sources other than glucose. The reduced size of mitochondria at the synapse, as mentioned above, critical for their inhabitance on synaptic terminals, can also translate in a greater flexibility at the level of fueling preference, particularly in the case of FAs [62]. To this extent, Ngo and colleagues showed a strong correlation between mitochondrial fragmentation and increased FAO rates (Figure 2). By interfering with the fusion and fission dynamics of mitochondria, the authors identified carnitine O-palmitoyltransferase 1 (CPT1) as the downstream effector of mitochondrial morphology in the regulation of FAO. With an increase in mitochondrial elongation, a higher CPT1 sensitivity to malonyl-CoA inhibition was observed, while inducing the fragmentation of mitochondria led to the reduced malonyl-CoA inhibition of CPT1, increasing long-chain but not short-chain FAO [62].

In summary, the topic of brain (and synaptic) metabolism needs to be further studied. As synaptic dysfunction generally precedes an unfavorable scenario of neuronal loss, understanding the metabolic fingerprint of synaptic terminals may be key to understanding which pathways go astray in the case of neurodegenerative conditions such as Alzheimer’s disease and Parkinson’s disease. To this extent, proper focus should be given to synaptic mitochondria, as these appear to be tailor-made to sustain the heavy demands imposed by the synaptic environment. The synapse may indeed harbor a unique flexibility on fuel usage that may have passed unnoticed to this day.
